# Xenotransplantation von Organen

**DOI:** 10.1007/s00104-024-02093-y

**Published:** 2024-05-15

**Authors:** Michael Schmoeckel, Matthias Längin, Bruno Reichart, Jan-Michael Abicht, Martin Bender, Joachim Denner, Georg Marckmann, Paolo Brenner, Eckhard Wolf, Christian Hagl

**Affiliations:** 1 Herzchirurgische Klinik und Poliklinik, LMU Klinikum – Standort Großhadern, Marchioninistr. 15, 81377 München, Deutschland; 2 Klinik für Anästhesiologie, LMU Klinikum Großhadern, München, Deutschland; 3 DFG-Sonderforschungsbereich TR127 – Xenotransplantation, LMU München, München, Deutschland; 4 Walter-Brendel-Zentrum für Experimentelle Medizin, LMU München, München, Deutschland; 5 Institut für Virologie, Fachbereich für Veterinärmedizin, FU Berlin, Berlin, Deutschland; 6 Institut für Ethik, Geschichte und Theorie der Medizin, LMU München, München, Deutschland; 7 Genzentrum und Center for Innovative Medical Models (CIMM), LMU München, München, Deutschland; 8 Partner Site München, Deutsches Zentrum für Herz- und Kreislaufforschung e. V. (DZHK), München, Deutschland

**Keywords:** Genetic Engineering, Organpräservation, Herz, Niere, Pilotstudie, Genetic engineering, Organ preservation, Heart, Kidney, Pilot study

## Abstract

Die Transplantation genetisch veränderter Schweineherzen und -nieren kann in den nächsten Jahren eine Lösung für den bestehenden Mangel an Organspendern darstellen. Fortschritte im Bereich des „Genetic Engineering“, aber auch verbesserte Organpräservationstechniken, eine Immunsuppression mit Kostimulationsblockade (Anti-CD40/CD40L-mAb) sowie eine verbesserte virologische Diagnostik, um eine Übertragung von pathogenen Schweineviren auf den Empfänger zu verhindern, haben hierzu beigetragen. Da Landrasse-Schweineorgane auch im Transplantatempfänger ihre Originalgröße erreichen, werden nun Schweinerassen verwendet, die entweder ein für den Menschen passendes Endgewicht erreichen (z. B. Auckland Island-Schweine) oder deren Wachstumshormonrezeptor genetisch inaktiviert wurde (z. B. in 10fach genetisch veränderten Schweinen der Fa. Revivicor/United Therapeutics, USA). Mit der ersten klinischen Pilotstudie an terminal Herzkranken wird in Deutschland in ca. 2 Jahren gerechnet.

**Graphic abstract:**

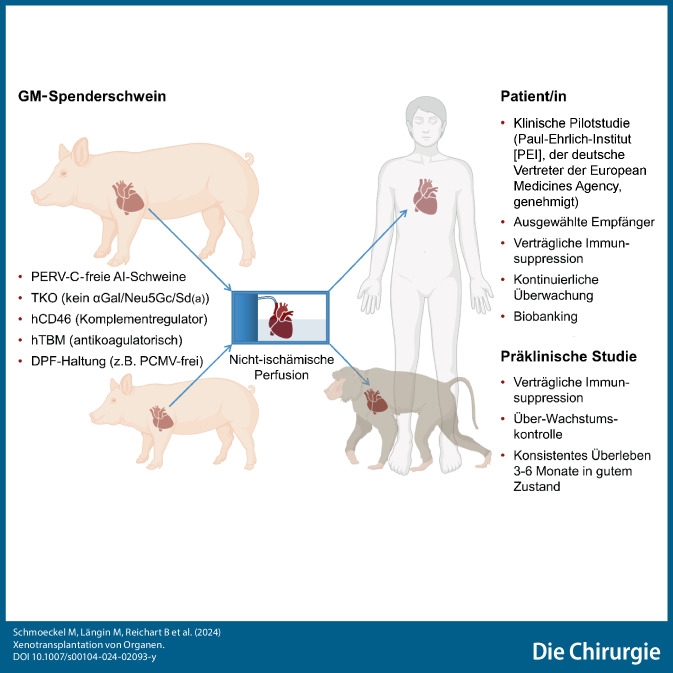

## Abstract (en)

Transplantation of genetically modified porcine hearts and kidneys could become a solution to the persistent shortage of human organ donors. Progress has been made in genetic engineering of donor pigs, preservation techniques after organ harvesting and immunosuppression using co-stimulation blockade with anti-CD40/CD40L monoclonal antibodies. Progress has also been made in in the development of methods that detect pathogenic porcine viruses and prevent their transmission to the recipient. As normal land breed pig organs continue to grow in the recipient to their original size, different pig breeds (such as Auckland Island pigs) are now used which reach a final size suitable for humans. Alternatively, a knock-out of the growth hormone receptor gene has been established, e.g., in the 10GM genetically modified pigs from Revivicor/United Therapeutics, USA. The first clinical pilot studies including patients suffering from terminal heart failure are expected to start in Germany in about 2 years.

In der vergangenen Dekade wurden im Bereich der Xenotransplantation erhebliche Fortschritte erzielt: Genetisch modifizierte (GM) Spenderschweine wurden generiert, Organkonservierungstechniken verbessert und wirksamere immunsuppressive Therapieregime entwickelt [1–5]. Ein Meilenstein wurde im Januar 2022 erreicht, als die erste klinische Schweineherzxenotransplantation bei einem Patienten mit terminaler Herzinsuffizienz in Baltimore, MD, USA, stattfand [6, 7]. Der Patient verstarb zwar nach 2 Monaten aufgrund der Übertragung eines Schweinevirus, des porzinen Zytomegalievirus, das zu einem Multiorganversagen und einer getriggerten Abstoßungsreaktion führte. Dennoch markierte dieser Heilversuch einen entscheidenden Schritt, um die klinische Machbarkeit der kardialen Xenotransplantation zu zeigen.

Im September 2023 wurde erneut an der University of Maryland in Baltimore eine zweite Xenoherztransplantation bei einem 58-jährigen Patienten durchgeführt, der aufgrund schwerwiegender peripherer Gefäßerkrankungen und Komplikationen mit inneren Blutungen nicht für eine allogene Herztransplantation oder ein Linksherz-Assist-Device (LVAD) infrage kam. Das Spendertier wies wiederum die gleichen 10 Genmodifikationen (10-GM) wie beim ersten Mal auf; der Patient verstarb nach 40 Tagen an einer akuten Abstoßungsreaktion.

Mitte 2022 erfolgten 3 orthotope Herztransplantationen in hirntoten Empfängern in New York (Langone Hospital) unter Verwendung derselben 10-GM-Schweine (United Therapeutics/Revivicor, Blacksburg, VA, USA). Die Herzen schlugen 72 h und 60 Tage lang ohne Anzeichen einer Abstoßung [8].

Mit dem gleichen Modell waren schon 1 Jahr zuvor xenogene Nierentransplantationen in Birmingham, AL, und wiederum in New York von unterschiedlich genetisch modifizierten Tieren erfolgt. Die Organe zeigten bis zu 54 h postoperativ eine normale Funktion. In New York konnte zum ersten Mal eine bioptisch gesicherte Abstoßungsreaktion einer nur einfach GM-xenogenen Transplantatniere (αGal-knock-out) erfolgreich therapiert werden [9].

Obwohl diese Experimente wertvolle Einblicke liefern, limitiert der instabile Zustand der hirntoten Empfänger längere Beobachtungszeiten [10, 11]. Zudem akzeptieren weder FDA (Food and Drug Administration) noch EMA (European Medicines Agency) die Ergebnisse für die Zulassung einer zukünftigen Pilotstudie am Menschen. Für aussagekräftigere Daten müssen daher präklinische Langzeitdaten an nichtmenschlichen Primaten (NMP) erbracht werden.

## Genetische Modifikationen der Spenderschweine zur Reduktion der Pathobiologie nach xenogener Transplantation

### Verhinderung von xenogenen Abstoßungsreaktionen

Die Komplexität der Pathobiologie bei der Xenotransplantation übertrifft bei Weitem die der Allotransplantation, da insbesondere die angeborene Immunantwort eine herausragende Rolle spielt [12–14]. Sowohl Menschen als auch nichtmenschliche Primaten (NMP) produzieren kurz nach der Geburt Antikörper, die auf das Kohlenhydratantigen „α(1,3)Gal“ reagieren, das auf der Oberfläche unveränderter Schweinezellen vorhanden ist. Folglich heften sich diese Antikörper schnell an die vaskulären Endothelzellen des Transplantats, wenn ein nicht modifiziertes Schweineorgan in einen Menschen oder Pavian transplantiert wird. Dies löst die Aktivierung der Komplementkaskade aus – bis hin zur Bildung von Membranangriffskomplexen C5b‑9 – und führt zur Infiltration von Leukozyten. Letztendlich kommt es zur Thrombosierung des Transplantats innerhalb von Minuten bis Stunden. Diese schnelle, von präformierten Antikörpern abhängige Abstoßung, wird als „hyperakut“ bezeichnet und ist durch histopathologische Merkmale wie venöse Thrombosen, Verlust der vaskulären Integrität, interstitielle Blutungen, Ödeme und die Infiltration von mononukleären Zellen gekennzeichnet [12].

Menschen haben im Gegensatz zu NMP neben Anti-α(1,3)Gal weitere präformierte Antikörper, nämlich gegen N‑Glycolylneuraminsäure (Neu5Gc) und ein Glykan, das dem humanen Sd(a)-Blutgruppenantigen ähnelt [15, 16].

Um die α(1,3)Gal‑, Neu5Gc- und Sd(a)-Epitope als Zielantigene für Abstoßungsreaktionen beim Menschen zu eliminieren, wurden Schweine mit inaktiven α‑1,3‑Galactosyltransferase(*GGTA1*)- [17], Cytidinmonophosphat-N-Acetylneuraminsäure-Hydroxylase(*CMAH*)- [18, 19] und β‑1,4‑N-Acetyl-Galactosaminyltransferase 2(*B4GALNT2*)-Genen [20] erzeugt, was zu den sog. „Triple-Knock-out(TKO)-Schweinen“ führte.

Die Komplementaktivierung kann jedoch auch über alternative Wege erfolgen, die nicht mit der Antikörperbindung zusammenhängen, wie beispielsweise durch einen Ischämie-Reperfusions-Schaden. Dies kann durch humane Komplementregulatoren (RCAs) verhindert werden – nämlich CD46 [21], CD55 [22] und CD59 [23]. Xenotransplantate von TKO-Tieren mit starker zusätzlicher Überexpression von einem oder mehreren humanen RCAs zeigten einen weitgehenden Schutz vor komplementvermittelten Abstoßungsreaktionen [14, 24, 25].

### Verhinderung von xenogenen Gerinnungsstörungen

Die Dysregulation der Gerinnung stellt einen weiteren Aspekt der Pathobiologie nach Xenotransplantation von Schweineorganen dar [26]. Sie wird von mehreren Faktoren beeinflusst, insbesondere durch die eben beschriebene Immunantwort, die eine Aktivierung des Gefäßendothels fördert und letztendlich zu einem prokoagulatorischen Zustand führt. Wesentlich ist aber auch die molekulare Inkompatibilität zwischen den Gerinnungsregulatoren von Schwein und NMP/Mensch.

Physiologisch bindet NMP/menschliches Thrombomodulin (TBM) auf Endothelzellen Thrombin aus dem Blutkreislauf. Dieser TBM-Thrombin-Komplex unterstützt die Bildung von aktiviertem Protein C, das eine starke antikoagulative Wirkung hat; Fibrinablagerungen in den Kapillaren werden somit verhindert. Demgegenüber kann porzines TBM zwar NMP/humanes Thrombin binden, aber nicht ausreichend Protein C aktivieren. Infolgedessen bilden sich massiv schädliche Fibringerinnsel im Kapillarsystem des Spenderorgans, was schließlich zur thrombotischen Mikroangiopathie führt.

Dies kann effektiv verhindert werden, indem Spenderschweine verwendet werden, die menschliches TBM auf ihren Endothelzellen exprimieren [27, 28].

### Nichtischämische Perfusionstechnik des Spenderschweineherzens

Über 2 Jahrzehnte hinweg waren die präklinischen Ergebnisse nach orthotopen xenogenen Herztransplantationen inkonsistent mit einer perioperativen Sterblichkeitsrate von 40–60 % [15, 29, 30]. Diese „perioperative kardiale Xenograftdysfunktion“ (PCXD) beruht auf einem Ischämie-Reperfusions-Schaden, denn Schweineorgane sind deutlich weniger resistent gegenüber einer Ischämie als menschliche Herzen. Seit 2015 wird die PCXD durch eine kontinuierliche, nichtischämische Perfusion der Transplantate mit einer kalten hyperonkotischen, oxygenierten kardioplegischen Lösung verhindert [31, 32]. Diese Konservierungstechnik wurde auch in den ersten klinischen Versuchen in Baltimore angewendet ([6, 7], persönliche Mitteilungen).

### Entwicklung eines nichtnephrotoxischen immunsuppressiven Regimes mit CD40- oder CD40L(CD154)-Kostimulationsblockade

Anfängliche Studien zur Schweineherztransplantation in Paviane verwendeten konventionelle immunsuppressive Regime ohne langfristigen Erfolg. Seit 2000 wurde die Kostimulationsblockade angewendet, zunächst mit einem monoklonalen Antikörper (mAb) gegen CD40L(CD154; [33, 34]). Aufgrund thrombotischer Komplikationen beim Menschen wurde er durch einen chimären Anti-CD40-mAb ersetzt, was erstmals zu längeren Überlebenszeiten führte [1, 3–5, 36]. In den klinischen Versuchen in Baltimore verwendete man humanisierte Versionen eines Anti-CD40- bzw. CD40L-Antikörpers zusammen mit Kortison und Mycophenolat-Mofetil ([6, 7], persönliche Mitteilungen).

### Kontrolle des schnellen Wachstums der Xenoorgane

Die für Xenotransplantationsversuche verwendeten Schweinerassen wie „Deutsche Landrasse“ oder „Large White“, wiegen ausgewachsen 200–300 kg und haben dann entsprechend große Herzen mit einem Gewicht von ca. 1 kg. Sie sind daher viel zu groß für einen menschlichen Empfänger, geschweige denn für einen Pavian. Neuere Erkenntnisse [1, 37] zeigten, dass das Wachstum des Spenderorgans genetisch reguliert ist: Das porzine Spenderherz verhält sich, als ob es sich noch im Körper eines schnell wachsenden Schweins befände. Zusätzlich führt der höhere Blutdruck bei den Pavianempfängern zu einer konzentrischen Hypertrophie des juvenilen Schweinemyokards. In Kombination bedingen diese Faktoren eine extreme Hypertrophie mit Entwicklung einer dynamischen linksventrikulären Ausflusstraktobstruktion [37].

Dieses überschießende Wachstumsphänomen wurde auch nach xenogener Nierentransplantation beobachtet [38, 39] und hat ebenso die Langzeitergebnisse limitiert.

Strategien zur Verhinderung der kardialen Größenzunahme umfassen in einem experimentellen Setting die medikamentöse Senkung des Blutdrucks, das frühzeitige Absetzen von Kortison und v. a. die Behandlung mit Sirolimus, einem ubiquitären Wachstumshemmer. Diese Maßnahmen reichen für Langzeiterfolge in präklinischen Experimenten aus.

Überschießendes Wachstum wird sowohl nach xenogener Herz- als auch Nierentransplantation beobachtet

**Abb. 1:**
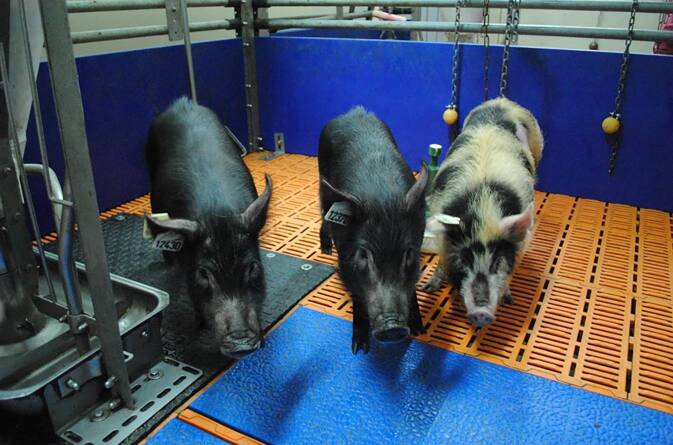
Auckland Island-Schweine im Tierversuchsgut der LMU, München-Oberschleißheim. (Mit freundl. Genehmigung, © LMU München, alle Rechte vorbehalten)

Für zukünftige klinische Anwendungen werden kleinere Spenderrassen benötigt, wie die Auckland Island(AI)-Schweine aus Neuseeland mit einem idealen Gewichtsbereich von 70–90 kg. Hierzu wurde eine kleine Herde von PERV-C- (s. unten) freien AI-Schweinen in der Nähe von München in einem Tierversuchsgut der LMU München etabliert (Abb. 1).

### Mikrobiologische Sicherheit

Eine Xenotransplantation kann mit der Übertragung von porzinen Mikroorganismen wie Viren, Bakterien, Pilzen und Parasiten verbunden sein [40–42]. Während Bakterien, Pilze und Parasiten einfach durch Zucht eliminiert werden können (oder antibiotisch behandelt werden), ist die Situation bei Viren komplizierter, aber lösbar.

In diesem Zusammenhang sei daran erinnert, dass auch bei Allotransplantationen menschliche Viren wie HSV (Herpes-simplex-Viren), CMV (Zytomegalievirus), EBV (Epstein-Barr-Virus), HIV (humanes Immundefizienzvirus), Tollwut oder Hepatitis B/C übertragen werden können – auch wegen der Kürze der Zeit, in der Explantationen stattfinden und die kein umfassendes Screening erlaubt. Im Gegensatz dazu können Schweine als Spender vor der Operation sorgfältig umfassend auf pathogene Viren untersucht werden. Xenogene Zoonosen sollten deshalb mit hoher Wahrscheinlichkeit zu vermeiden sein.

Wichtig ist das Hepatitis-E-Virus Genotyp 3 (HEV3), ein gut untersuchtes zoonotisches Virus, das durch den Verzehr von unzureichend gegartem Schweinefleisch oder durch Kontakt mit Schweinen auf den Menschen übertragen wird. Bei Immunsupprimierten können chronische Infektionen verursacht werden, bestehende Lebererkrankungen verschlimmern sich [43, 44].

Ein Herpesvirus, das porzine Zytomegalievirus – eigentlich ein porzines Roseolovirus (PCMV/PRV) –, ist ein weiterer, gefährlicher Mikroorganismus. PCMV/PRV ist eng mit den menschlichen Herpesviren 6 und 7 verwandt [45], also nicht mit dem humanen CMV, das nach Allotransplantationen schwerwiegende Lungenkomplikationen verursacht [46]. Bis vor Kurzem wurde gezeigt, dass PCMV/PRV nur für NMP schädlich ist: Die Übertragung des Virus auf Paviane und Rhesusaffen verringerte signifikant die Überlebenszeit der Xenotransplantate [47, 48]. Aber auch bei der ersten Transplantation eines 10-GM-Schweineherzens auf einen Menschen in den USA wurde PCMV/PRV übertragen und trug offensichtlich zum Tod des Empfängers bei [6, 7]. Das Virus interagierte dabei wohl direkt mit seinem Immunsystem und den Endothelzellen. Die virale Sicherheit entscheidet demnach wesentlich über den Erfolg von Xenotransplantationen, und es müssen daher entsprechend sensitive Untersuchungsmethoden zum Ausschluss verwendet werden [49].

Da es gegen PCMV/PRV keine Vakzine oder Medikamente gibt, kommt in München eine präventive Strategie zum Einsatz: PCMV (und HEV [Hepatitis-E-Virus]) werden durch frühzeitiges Entwöhnen der Ferkel eliminiert, d. h. die Ferkel werden nicht mehr gesäugt, da sonst die Viren über die Schnauze der Mutter übertragen werden können. Die Tiere werden nach der Geburt per Sectio in eine DPF(„designated pathogen free“)-Unit verbracht und unter gezielt keimfreien Bedingungen aufgezogen [50].

Die virale Sicherheit entscheidet wesentlich über den Erfolg von Xenotransplantationen

Diese Strategie kann jedoch nicht porzine endogene Retroviren (PERVs) eliminieren, da diese in das Genom aller Schweine integriert sind [51]: PERV‑A und -B sind in allen Schweinen nachweisbar, und können menschliche Zellen in vitro (unter speziellen experimentellen Bedingungen) infizieren. PERV‑C ist nicht in allen Schweinen vorhanden und infiziert nur Schweinezellen. Allerdings kann PERV‑C mit PERV‑A rekombinieren, und die resultierenden Rekombinanten können menschliche Zellen infizieren [52–54]. Deshalb wurden für München Auckland Island-Schweine ausgewählt, die PERV-C-frei sind [55].

Festzuhalten bleibt, dass die Übertragung von PERVs in keiner der vielen klinischen und präklinischen Xenotransplantationsstudien, die bisher durchgeführt wurden, und in keinem der experimentellen PERV-Infektionsversuche beobachtet wurde [56].

## Patientenauswahl für eine xenogene Herztransplantation

Die Auswahl der ersten Patienten für klinische Studien zur kardialen Xenotransplantation erfordert sorgfältige Überlegungen, um die inhärenten Risiken zu rechtfertigen und mit hoher Wahrscheinlichkeit günstige Ergebnisse zu gewährleisten [57, 58].

Mögliche erste Kandidaten könnten ältere Patienten > 70 Jahre oder intensivpflichtige Personen sein, die für eine mechanische Kreislaufunterstützung (MCS) aufgrund einer Antikoagulanzienunverträglichkeit nicht geeignet sind. Darüber hinaus kommen terminal Kranke mit (degenerierten) Bio- oder mechanischen Herzklappenprothesen, hypertropher Kardiomyopathie, biventrikulärem Herzversagen oder einer Postinfarkt-Ventrikelseptum-Ruptur infrage [59]. Diese Patienten werden auch aufgrund ihrer Abhängigkeit von inotropen Medikamenten und den dann vorhandenen Arrhythmien instabil [35].

Neugeborene und Säuglinge mit komplexen angeborenen Herzkrankheiten – wie z. B. dem hypoplastischen Linksherzsyndrom – können aufgrund des Mangels an Spendern und der schlechten Ergebnisse an einer MCS [60] oder auch nach dem Versagen einer Fontan-Operation – am meisten von einer kardialen Xenotransplantation profitieren. Auch eine nachfolgende Allotransplantation („bridge to allotransplantation“) ist vorstellbar.

Ein Vorteil bei Neugeborenen wäre darüber hinaus ihr noch unreifes Immunsystem. In Kombination mit einer Thymektomie/Spenderthymustransplantation zum Zeitpunkt des Eingriffs könnten dies Voraussetzungen für eine operative Toleranz sein [61].

## Patientenauswahl für eine xenogene Nierentransplantation

Während nach einer Herztransplantation eine ausreichende Pumpfunktion des Transplantats ganz im Vordergrund steht, erfordert die xenogene Nierentransplantation – neben der suffizienten Ausscheidung harnpflichtiger Metabolite – auch eine Funktionalität endokriner Systeme wie die des Renin-Angiotensin-Aldosteron-Systems (RAAS), die Homöostase von Kalzium, Phosphat, Vitamin D und dem Parathormon und die von Erythropoietin. Trotz einer Speziesinkompatibilität von Renin war in präklinischen NMP-Experimenten jedoch kein gestörter Flüssigkeitshaushalt zu beobachten. Bislang war in diesen Studien lediglich ein Trend zu erhöhten Kalziumspiegeln zu messen. Erythropoetin steht als rekombinantes humanes Medikament zur Verfügung und kann daher – falls nötig – substituiert werden. Vorteilhaft ist, dass – anders als beim Menschen – eine porzine Niere auch in der Lage ist, Harnsäure auszuscheiden [62].

Für den Beginn klinischer Xenotransplantationen werden v. a. Patienten mit Shuntproblemen infrage kommen, aber auch HLA(humanes Leukozytenantigen)-Hypersensibilisierte. Bei Letzteren werden Cross-Match-Tests entscheidend sein, um bestehende Anti-SLA(„swine leukocyte antigen“)-Antikörper zu detektieren (z. B. mit Tests wie „complement dependent cytotoxicity“ [CDC], [63–67]).

## Fazit für die Praxis

- Multiple Modifikationen des Schweinegenoms, neue Organpräservationstechniken, die Immunsuppression mit Kostimulationsblockade, kleinere Spenderspezies und effiziente Virusnachweismethoden haben es ermöglicht, ein Langzeitüberleben von Schweineherzen und -nieren nach Transplantation in nichtmenschliche Primaten zu erzielen.
- Die Gefahr einer Übertragung von Infektionen kann durch Auswahl entsprechender Rassen, sensitive Assays und Aufzucht in einer DPF(„designated pathogen free“)-Unit ausgeschlossen werden.
- Zusammenfassend ist davon auszugehen, dass klinische Xenotransplantationen in den nächsten Jahren bedeutende Fortschritte machen und die diejenigen einer mechanischen Kreislaufunterstützung, der Stammzellübertragung und regenerativen Medizin übertreffen werden. Mit dem Beginn erster klinischer (von FDA [Food and Drug Administration] und EMA [European Medicines Agency] zugelassener) Studien wird in den nächsten Jahren gerechnet.
- Voraussetzung für die erfolgreiche Durchführung sind Teams, die sich mit der Methode der Xenotransplantation präklinisch erfolgreich beschäftigt haben.
